# Influence of Stent Structure on Mechanical and Degradation Properties of Poly (Lactic Acid) Vascular Stent

**DOI:** 10.3390/jfb16070248

**Published:** 2025-07-06

**Authors:** Shicheng He, Qiang Chen, Zhiyong Li

**Affiliations:** 1 Biomechanics Laboratory, School of Biological Science & Medical Engineering, Southeast University, Nanjing 210096, China; 230198592@seu.edu.cn; 2 School of Mechanical, Medical and Process Engineering, Queensland University of Technology, Brisbane, QLD 4001, Australia; 3 Faculty of Sports Science, Ningbo University, Ningbo 315211, China

**Keywords:** biodegradable vascular stents, finite element analysis, radial stiffness, degradation property

## Abstract

Biodegradable vascular stents (BVSs) face challenges related to inadequate mechanical strength, which can lead to adverse clinical outcomes. Improving the mechanical behavior of biodegradable vascular stents through structural design has been extensively explored. However, the corresponding effects of these mechanical enhancements on degradation characteristics remain under-investigated. The present work focuses on examining how different stent design strategies affect the mechanical behavior and degradation characteristics of poly (lactic acid) (PLA) stents. The commercial PLA stent DESolve was adopted, and nine modified stents were constructed based on the geometrical configuration of the DESolve stent. The mechanical properties of the modified stents during radial crimping and three-point bending simulations were thoroughly studied. The degradation dynamics of the stents were characterized by four indices (i.e., mean number average molecular weight, residual volume fraction, mean von Mises stress, and stent diameter). The results indicated that both the widening ratio and direction affected the mechanical performance of the stents by increasing the radial stiffness and radial strength, minimizing recoil%, and decreasing the bending flexibility. Although the widening direction had a relatively minor influence on stent degradation, the associated increase in material volume contributed to an improved volumetric integrity and enhanced lumen preservation. This study established a theoretical basis for evaluating both the mechanical and degradation behaviors of PLA stents, offering valuable insights for future structural design optimization.

## 1. Introduction

Biodegradable vascular stents are deemed as promising implantable medical devices for the treatment of atherosclerotic diseases due to their superior short-term mechanical support and long-term biodegradable properties [1,2]. Recent advances in biodegradable stent technology have explored both metallic and polymeric materials, each with distinct characteristics. Metallic options such as magnesium alloys (e.g., WE43) and zinc-based systems demonstrate a superior radial strength but face challenges with rapid degradation and potential hydrogen gas accumulation [3,4,5,6]. Polymers, including poly(ε-caprolactone) (PCL) and poly (glycolic acid) (PGA), offer tunable degradation rates, yet PCL’s slow resorption (>3 years) and PGA’s acidic degradation byproducts limit their clinical utility [7,8]. As one of the most favorable biodegradable vascular stents, poly (lactic acid) (PLA) stents exhibit a commendable biocompatibility. However, the mechanical behavior of PLA stents is significantly inferior to that of traditional metallic stents, and this is attributed to the intrinsic low mechanical properties of PLA materials [9]. The poor mechanical properties of stents pose significant clinical challenges. Inadequate radial strength and excessive elastic recoil may lead to serious complications, such as acute stent collapse, late discontinuity, and stent malapposition [10]. These complications can further result in severe clinical symptoms, including myocardial infarction and stroke [11]. More importantly, the in vivo degradation of a PLA stent is uncontrollable due to the complex in vivo mechanical environment and the stress distribution of the stent. Therefore, it is imperative to enhance the mechanical behavior of PLA stents and effectively predict the degradation behavior of these stents in order to improve their clinical efficacy and safety.

The insufficient radial strength and excessive elastic recoil of PLA stents are the main concerns in clinical applications. Thickening the stent strut has been proven to be an efficient way of increasing radial stiffness and reducing elastic recoil% [12], but a thicker stent strut results in a narrowing vascular lumen and further impedes blood flow, thus leading to in-stent restenosis [13]. Recently, many researchers have concentrated on stent structural design to improve the mechanical properties of PLA stents [14,15,16,17]. For example, Wei et al. [18] developed a novel type of bioresorbable stent called the open-cell stent (OCS) and conducted a finite element analysis to compare the OCS with the commercial Abbott BVS 1.1. The key findings revealed that the radial strength and recoil%of the OCS were enhanced by 30% and 24%, respectively. Wang et al. [14] designed three PLA stents with different configurations and systematically evaluated their mechanical properties. The study demonstrated that the geometric design of a stent significantly influences the overall mechanical performance. In addition, the radial stiffness and bending stiffness of the stents were also studied [19,20], as they are associated with expansion ability and compliance during stent deployment. The findings suggested that critical performance parameters such as recoil%, bending flexibility, and radial stiffness often exhibit inherent conflicts, in other words, improvements of these parameters are trade-off. Although previous extensive studies have focused on enhancing the mechanical properties of stents, they rarely addressed the degradation behavior of PLA stents, which is an inherent problem when designing degradable PLA stents. This is because after PLA stent deployment, the in vivo degradation of PLA stents plays a more important role in maintaining their long-term efficacy and safety in clinical applications [21].

Addressing the structure and degradation of PLA stents, the authors recently investigated the different mechanical and degradation behaviors of four typical commercial PLA stent devices (i.e., Absorb, DESolve, Igaki-Tamai, and Fantom) based on a three-factor regulated degradation model and proved that the DESolve stent was the best according to a pilot scoring system composed of radial stiffness, degradation, residual volume, mean stress level, and final diameter [22]. It is worth mentioning that the DESolve stent still has limitations, such as excessively rapid localized degradation at the ring bend positions. Moreover, the rapidly localized degradation observed at these bending positions was also found in other biodegradable stents, such as the zinc alloy stent [23,24]. The reason for the fast degradation at these positions is attributed to their high stress levels, which facilitate material degradation. Thus, considering that thickening the stent strut was proven to be an efficient way to increase radial stiffness and reduce elastic recoil% [12], but a thicker stent strut resulted in a narrowing vascular lumen and further impeded blood flow, thus leading to in-stent restenosis [13], widening the stent strut may be an alternative option.

To this end, this study proposed three ring bend stent configurations by modifying the geometry to reduce rapidly localized degradation and numerically evaluated the effects of the configurations on mechanical and degradation performances based on the optimal commercial DESolve stent in our previous study. The ring bends of the stents were widened in three ways by three widening ratios. Radial crimping and three-point bending simulations were conducted to characterize the radial mechanical properties and bending flexibility of the stents. By employing the previously developed PLA degradation model, the stent degradation processes were determined and the degradation properties of the modified stents were characterized with four indices (i.e., the mean number average molecular weight, the residual volume fraction, the mean von Mises stress, and the stent diameter). Finally, the influence of the modified stent structure on the mechanical and degradation properties was discussed, and the best widening method for the stent configuration was suggested.

## 2. Methods

### 2.1. Widening Strategy for the Ring Bend

**Figure 1:**
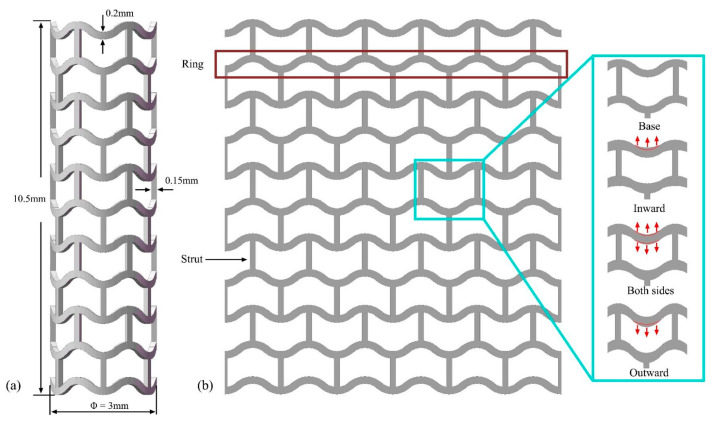
Geometrical configurations of the designed stent. (**a**) Stent geometric parameter definitions and (**b**) two-dimensional sketch of the stent and three widening strategies.

A stent is composed of two components, i.e., the supporting ring and connecting strut. The former is responsible for lumen expansion and the latter for bending flexibility. The commercial DESolve stent was adopted, and its geometrical parameters were defined according to the literature [25]. The stent was composed of eleven supporting rings and ten rows of connecting struts, which were distributed uniformly in the circumferential direction to connect the rings, as shown in Figure 1. The initial length and diameter of the stent were 10.5 mm and 3 mm, respectively, and the width and thickness of the strut were 0.2 mm and 0.15 mm, respectively. To mitigate rapid degradation at the stent’s ring bends, we implemented three widening strategies (inward, both-side, and outward) by increasing the bend width by 10%, 20%, and 40% relative to the base width 0.2 mm. The widening directions and initial stent boundary are delineated by arrows and red contours in the Figure 1b inset. Notably, the typical strut width of commercial stents (0.15–0.2 mm) falls precisely within the 40% widening ratio threshold established in this study (0.2–0.28 mm) [2,26]. Therefore, nine modified stents were obtained, and the ring bend width ranged from 0.22 mm to 0.28 mm. To maintain consistent terminology while reflecting design modifications, we designated the stent variants as Base (unmodified DESolve stent), In-X%, Out-X%, and Both-X% (inward, outward, and both-side widening by X%, where X = 10, 20, or 40). All variants are detailed in Table S1 with dimensional parameters.

### 2.2. Degradation Model of PLA

The degradation of PLA was detailed in our previous study [22]. Briefly, its degradation rate was governed by mechanical stress, autocatalysis, and swelling based on the polymer hydrolysis mechanism. Two degradation criteria were used to judge the complete degradation of PLA material points, and they were based on the threshold of the normalized number average molecular weight and the occurrence of the stochastic event of polymer chain breakage. Moreover, the ideal elastic–plastic material model was used to define the PLA. In this regard, the reduced Young’s modulus of PLA material points is correlated to the normalized number average molecular weight. The yield strength of PLA materials is influenced by the combined effects of degradation and fatigue, which were characterized by an exponential decay model (degradation-rate-dependent) and a strength reduction coefficient, respectively.

### 2.3. Finite Element Analysis

In the present finite element analysis (FEA), we performed (1) crimping and bending simulations of the ten whole stent models to examine their radial stiffness and flexibility, respectively, and (2) degradation simulations of their stent segments interacting with the vessel and balloon to evaluate their degradation kinetics thanks to the periodicity of the stent.

The stent meshing strategy was also adopted from our previous studies [22,27]. Moreover, a mesh sensitivity study was also carried out prior to all simulations to ensure that a balance between computational efficiency and numerical accuracy was achieved for both the mechanical and degradation analyses. This analysis revealed minimal variation (<5%) in the radial stiffness of the DESolve stent during crimping simulations. These findings informed the selections of both the optimal mesh type and element size for the current study [27]. In the crimping and bending analyses, each of the ten whole stent models was discretized using four-node linear tetrahedral elements (C3D4), with the total element count ranging from 177,809 to 216,021. The average element size was maintained at 0.06 mm. For the degradation simulation, hexahedral elements (C3D8R) were employed for both the stent and vessel, while the balloon structure was meshed using shell elements (S4R). In this setup, each stent segment contained between 10944 and 14976 elements with a uniform size of 0.05 mm. The vessel and balloon meshes consisted of 13200 and 1590 elements, respectively, each with a size of 0.12 mm. A summary of the mesh statistics for the ten whole stent models and corresponding segments in both simulations is provided in Table S2. Unlike the PLA stent, which followed an elastoplastic material description, both the vessel and balloon were treated as incompressible, isotropic, and hyper-elastic materials, modeled using a two-parameter Mooney–Rivlin formulation [27,28], as follows:

$$W=C_{10}\left({\bar{I}}_{1}-3\right)+C_{01}\left({\bar{I}}_{2}-3\right)$$

where $C_{10}$ and $C_{01}$ are material constants, and ${\bar{I}}_{1}$ and ${\bar{I}}_{2}$ are the first and second strain invariants, respectively. The base material parameters can be found in Table 1.

#### 2.3.1. Simulation of Stent Crimping

**Figure 2:**
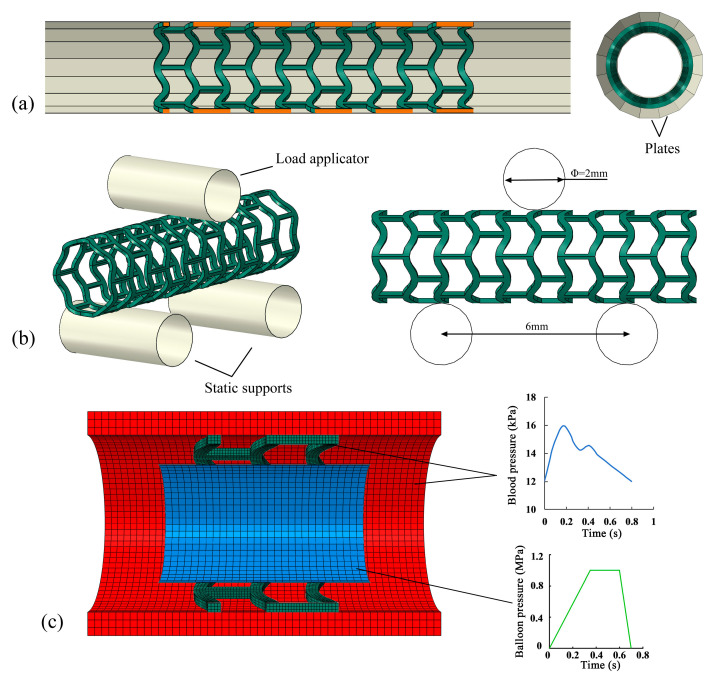
FEM models. (**a**) The stent’s crimping model and plate distributions, (**b**) three-point bending model, and (**c**) the stent–balloon–vessel assembly system and loading conditions, subfigures a and c are reprinted from [22].

The crimping process was simulated by uniformly positioning sixteen rigid plates around the stent to exert radial compression (Figure 2a), with each plate measuring 30 mm in length and 2 mm in width. A radial displacement of 0.8 mm was loaded on the rigid plates to compress the stent’s outer diameter from 3.0 mm to 1.4 mm. After that, the rigid plates were unloaded to their initial position. The boundary conditions were defined by fixing one end of the stent to restrict it to radial deformation, while leaving the other end unrestrained to permit axial extension. Throughout the crimping simulation, the radial displacement and corresponding reaction forces of the rigid plates were monitored, and subsequently, the force–diameter relationship was established.

#### 2.3.2. Simulation of Stent Bending Flexibility

The three-point bending test quantitatively assessed stent flexibility, a critical determinant of clinical conformability. An enhanced bending performance enables improved adaptation to vascular curvature, reducing vessel straightening effects during deployment. Since the present study focuses on the mechanical and degradation properties of geometrically modified stents, it is necessary to include bending test simulations even though the stents hardly experience bending during degradation. The three-point bending simulations of the stents followed the ASTM F2606–08 standard [32]. In the model, two fixed supports and a central load applicator were represented as rigid cylinders, each with a diameter of 2 mm and a length of 5 mm. Considering a stent length of 10.5 mm, the suggested span length between the two supports was no more than 6.7 mm, and deflection of no more than 1.2 mm was allowed (Figure 2b). In the simulation, a downward displacement of 1.0 mm was applied on the load applicator to bend the stent to guarantee a deflection of less than 1.2 mm. Then, the load applicator was removed to the initial position. The reaction force and displacement of the load applicator were recorded, and a bending force–deflection curve was obtained.

#### 2.3.3. Simulation of Stent Degradation

As illustrated in Figure 2c, the vessel geometry was simplified as a straight cylindrical tube, measuring 6 mm in length, with an internal diameter of 3.0 mm and a wall thickness of 0.4 mm. Similarly, the balloon was represented by a simplified tubular structure, featuring a length of 3.6 mm, an internal diameter of 2.0 mm, and a wall thickness of 0.06 mm. To reduce computational cost, a stent segment consisting of three repeating support rings was selected for the degradation analysis [17,23,24,33]. Initially, an internal pressure (indicated by the green line in Figure 2c) was exerted on the balloon’s inner surface to dilate both the stent and the vessel. Following balloon deflation, a physiologically representative blood pressure waveform (blue line in Figure 2c) was applied to the inner surfaces of the stent and vessel to induce cyclic loading, thereby initiating the degradation process. To approximate in vivo boundary conditions, both ends of the vessel were fixed. Additionally, six nodal points on the central support ring of the stent were restricted to only radially move, while its two ends were left unconstrained. Contact interactions between the stent and compression plates, balloon and stent, and stent and vessel were all modeled using a penalty-based formulation with a friction coefficient of 0.1. The mechanical response and degradation behavior were simulated using ABAQUS/Explicit, where the degradation model was implemented through a user-defined VUMAT subroutine. All relevant input parameters are detailed in our previous publication [22].

## 3. Results and Discussion

### 3.1. Radial Mechanical Performances of Stents

**Figure 3:**
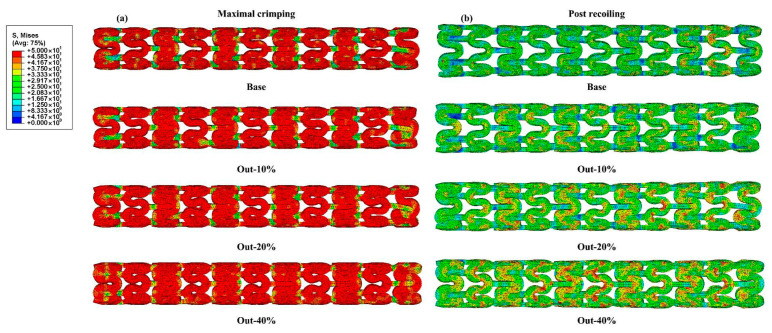
The von Mises stress of the Out-modified stents in (**a**) the maximal crimping and (**b**) the post-recoiling states.

Figure 3 shows the von Mises stress distributions of the representative stents (Base, Out-10%, Out-20%, and Out-40% stents) during the crimping simulation. All of the stents uniformly deformed without rotation or breakage. Overall, the four stents exhibited a similar stress distribution when crimped to the minimum diameter of 1.4 mm, with higher stress concentrated in the supporting rings and lower stress in the connecting struts. This is because the supporting rings underwent greater deformation, resulting in higher stress [34]. Compared to the Out-40% stent, the stress on the connecting struts of the other three stents was lower, indicating a reduced volume of yielded PLA in these stents. In the post-recoiling state, all stents underwent uniform radial deformation, leading to a significant reduction in stress levels compared to the maximal crimping state. Moreover, as the widening ratio increased, the area of the high-stress region also increased. This was consistent with the higher volume of yielded PLA in the widened stents, which resulted in greater stress after recoiling.

**Figure 4:**
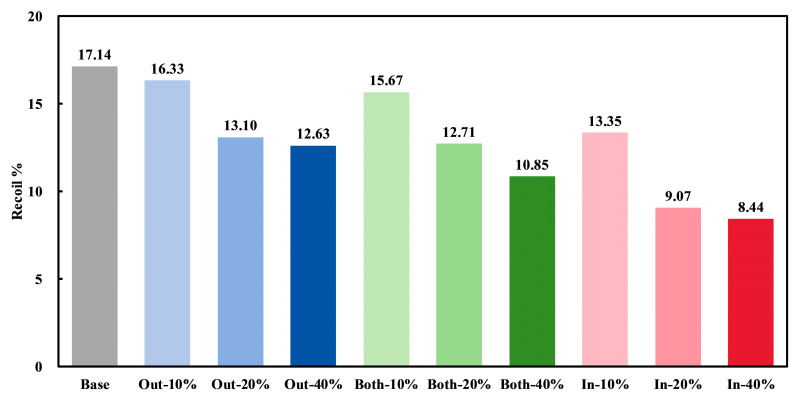
Recoil% of the simulated stents.

The stent diameters in the maximal crimping state (Dcrimp) and post-recoiling state (Drecoil) were measured and are listed in Table S3. The recoil% of all stents after crimping was calculated using the equation |Dcrimp−Drecoil|/Dcrimp × 100%, as shown in Figure 4. It was observed that the radial recoil% of all widened stents was lower than that of the Base stent. This reduction can be attributed to the increased material volume in the widened stents, which resulted in a larger region of yielded PLA under identical radial compression (Figure 3). Among all the stents, the In-40% stent exhibited the lowest recoil% of 8.44%, which is comparable to the crimping recoil of PLA stents, which ranges from 5% to 30% [35,36]. For any widening ratio, the Out-modified stents had the highest recoil%, followed by the Both-modified stents, while the In-modified stents exhibited the lowest recoil%. Moreover, as the widening ratio increased, the radial recoil% of the stents decreased for all widening directions. This indicates that the wider the stent, the lower its radial recoil%. Interestingly, the recoil% of the In-20% stent was the second lowest with a value of 9.07%, much lower than that of the Out-40% and Both-40% stents. This indicates that although both the widening direction and widening ratio reduced the stent’s recoil%, their impacts differed in magnitude. This suggests that specific parameter combinations should be considered in the structural design of stents to achieve the desired characteristics.

**Figure 5:**
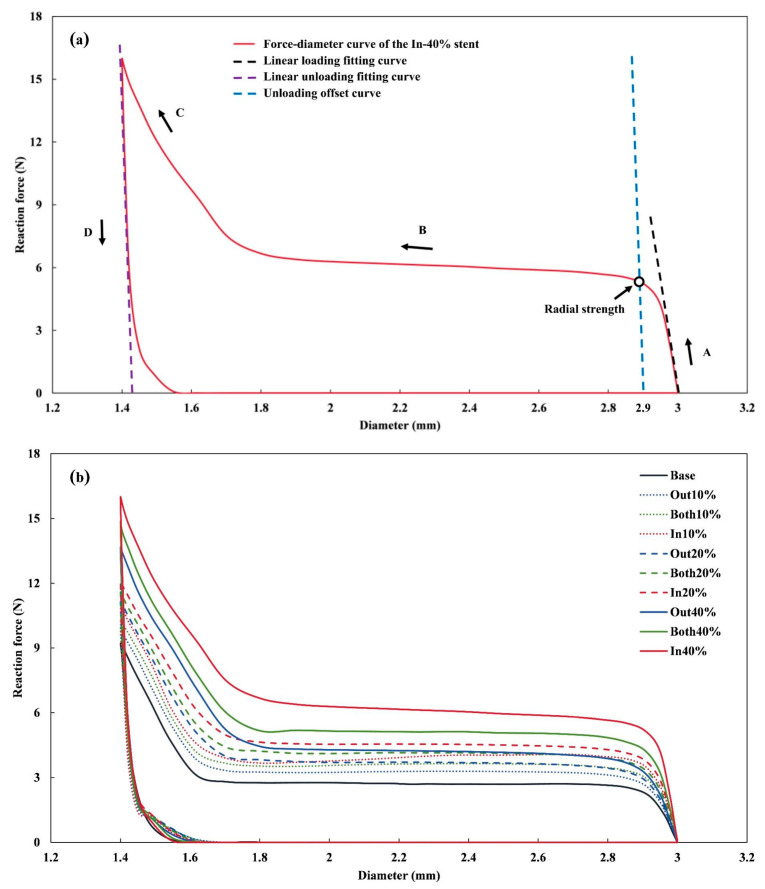
Force–diameter curves of the modified stents during the entire crimping process. (**a**) Different stages during crimping and (**b**) force–diameter curves of all stents.

Figure 5 illustrates the relationship between the reaction force of the rigid plates and the outer diameter of the stents during the crimping analysis. All stents exhibited a similar force–displacement pattern, which can be segmented into the following four distinct phases: (A) elastic loading, (B) increasing plasticity, (C) plastic strengthening, and (D) elastic unloading, consistent with previous reports [37]. In the initial loading phase, stent deformation remained minimal, and a linear correlation was observed between compressive force and displacement within the elastic range. As the compressive displacement continued to increase, plastic deformation began once the stress exceeded the material’s yield strength, leading to a nonlinear force–displacement response. Following the ASTM F3067 guideline for stent crimping [11], the slope of the linear elastic loading curve was used to determine the stent’s radial stiffness. Radial strength was calculated based on a 3% reduction in diameter, corresponding to an unloading offset of 0.09 mm [15]. The unloading offset curve was defined by the slope of the linear unloading segment and an X-axis intercept of 2.91 mm. The intersection point between the loading and unloading offset curves was identified as the stent’s radial strength. A summary of the radial stiffness and strength values for all stent designs is presented in Table 2. The In-40% stent demonstrated significant improvements in both radial stiffness (83.72 N/mm) and radial strength (0.49 N/mm), with increases of 143.94% and 104.17% compared to the Base stent, respectively.

### 3.2. Bending Stiffness of Stents

**Figure 6:**
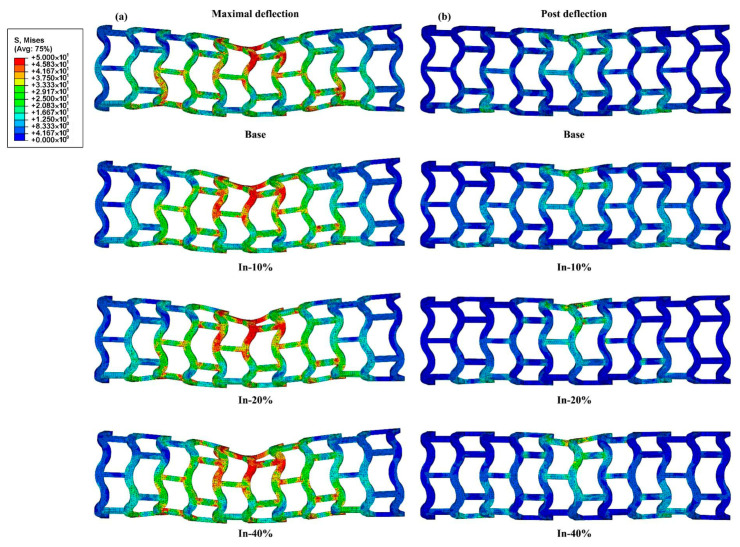
The von Mises stresses of the In-modified stents in (**a**) the maximal deflection and (**b**) the post-deflection states.

Figure 6 shows the von Mises stress distributions of the representative stents (Base, In-10%, In-20%, and In-40% stents) during the bending simulation. Generally, all stents exhibited a similar stress distribution in both states. High stress concentrations were observed in areas where the load applicator contacted with the stent, corresponding to regions that exhibited significant deformation during the bending simulation. The In-40% stent exhibited the highest stress concentration when deflected to 1 mm, indicating that it experienced greater forces compared to the other configurations. In the post-deflection state, the stress levels of all stents decreased significantly, which indicates that elastic deformation dominated in the bending simulation [38].

**Figure 7:**
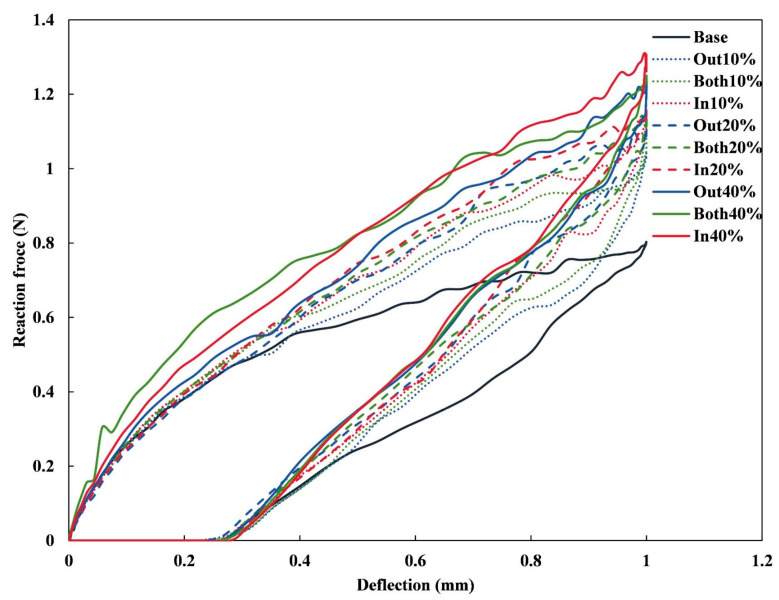
Three-point bending force–deflection curves of the simulated stents.

The force–deflection responses of all stents under three-point bending are depicted in Figure 7. The Base stent showed the lowest reaction force across the range of deflections, indicating its relative flexibility compared to the other stents. In contrast, the In-40% stent exhibited the highest reaction force, suggesting a greater resistance to deformation, which aligns with its enhanced radial stiffness. The other configurations (e.g., Out-10% and Both-20%) fell between the two extremes, indicating varying levels of stiffness and strength based on the widening direction and ratio. According to the formulation introduced in previous studies [39], bending stiffness is determined by a function of span length, deflection, and applied force, and is commonly used to assess a stent’s resistance to bending-induced deformation. Herein, the bending stiffness was calculated using the classical expression $\mathrm{bending}\;\mathrm{stiffness}=\frac{FL^{3}}{48f}$, where *F* denotes the concentrated load, *L* is the span length, and *f* represents the corresponding bending deflection. With a deflection value set at 1 mm, the bending stiffness values for all stent configurations were computed and are summarized in Table 3. The results reveal that, under identical widening ratios, the In-modified stents displayed a higher bending stiffness. Moreover, an increasing trend in bending stiffness was observed as the widening ratio increased. Specifically, the In-40% stent exhibited the highest bending stiffness, with a value of 5.90 N·mm^2^, marking a significant enhancement of 63.43% compared to the Base stent’s stiffness of 3.61 N·mm^2^. In contrast, the Out-10% stent demonstrated a flexural stiffness of 4.68 N·mm^2^, representing the lowest increase of 29.64%.

It is worth mentioning that during stent design, a balance between bending stiffness and flexibility must be achieved [40,41]. For applications requiring a higher radial strength (e.g., stents implanted in large vessels), high-stiffness stents (such as the In-40% stent) can provide greater support, thereby effectively maintaining vascular lumen morphology. Conversely, for scenarios requiring adaptation to complex vascular curvatures (e.g., curved or bifurcated vessels), more flexible stents (such as the Out-10% stent) have advantages.

**Figure 8:**
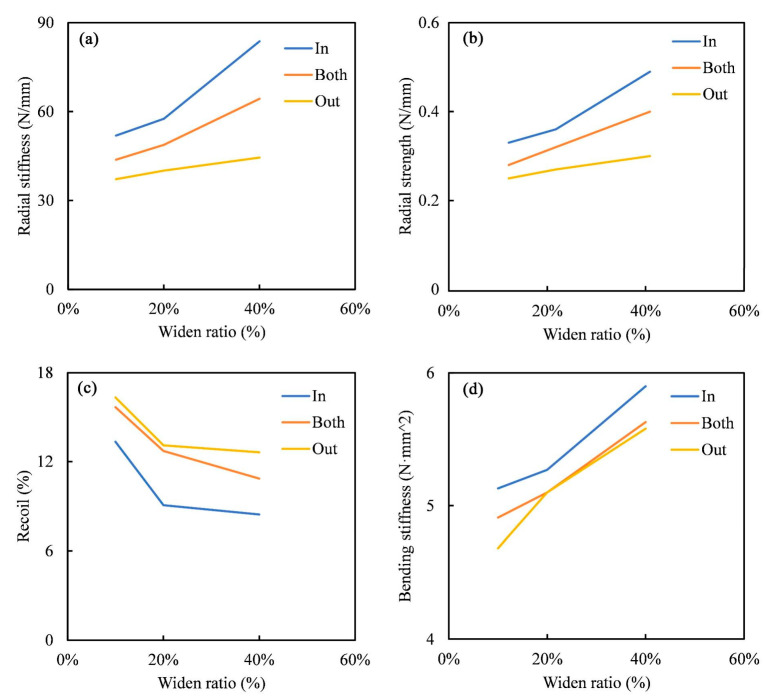
Impact of widening ratios on stent mechanical performances. (**a**) Radial stiffness, (**b**) radial strength, (**c**) radial recoil%, and (**d**) bending stiffness.

To clearly illustrate the impact of widening directions and ratios on the mechanical performances of the stents, Figure 8 presents the radial stiffness, radial strength, recoil%, and bending stiffness of all stents in relation to the widening ratio. With an increase in the widening ratio, the radial stiffness, radial strength, and bending stiffness of the stents significantly increased in all widening directions, while the radial recoil% decreased markedly. This indicates that a higher widening ratio substantially enhanced the stents’ radial support performance. Under a fixed widening ratio, the In-modified stents exhibited the optimal radial support performance. Furthermore, for stents with different widening directions, the improvement in radial support performance demonstrated a nonlinear trend with increasing widening ratios. Taking the In-modified stents as an example, after the widening ratio exceeded 20%, the increasing rates of radial stiffness, radial strength, and bending stiffness accelerated, while the decline in radial recoil% slowed. These results suggest that locally widening the support rings can effectively enhance a stent’s radial support performance, with a more pronounced effect at higher widening ratios. However, as the widening ratio increased, the stents’ porosity and flexibility decreased accordingly, which may adversely affect their deliverability and adaptability to curved vessels [42,43]. Therefore, the widening direction and ratio should be carefully selected based on mechanical requirements to balance the radial support ability and flexibility, thereby avoiding potential adverse clinical outcomes. These mechanical modifications also significantly influence the following degradation behaviors.

### 3.3. Degradation Properties of Stents

The degradation properties of the stents were characterized by four indices, including the mean number average molecular weight $\bar{\beta }\left(t\right)$, the residual volume fraction of the stent $v_{r}\left(t\right)$, the mean von Mises stress $\bar{\sigma }\left(t\right)$, and the stent diameter D(t), by referring to the author’s previous research [22]. Herein, the impacts of both the widening ratio and widening direction on stent degradation are discussed in the following sections.

**Figure 9:**
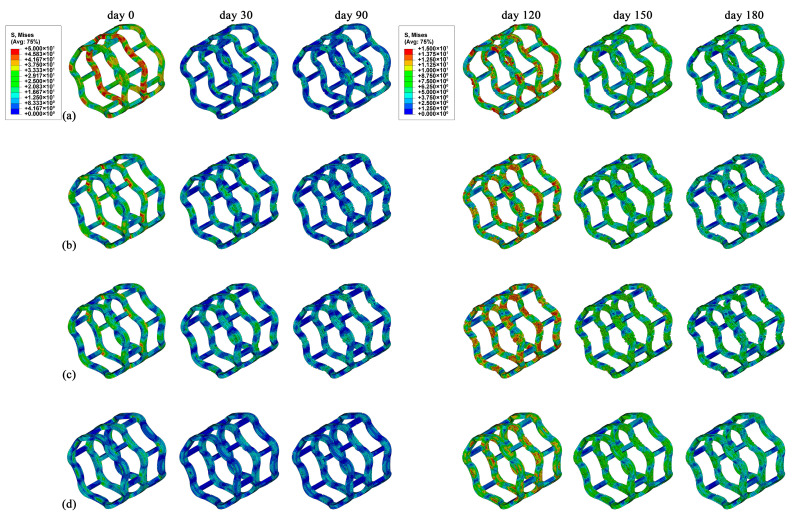
The von Mises stress distributions of the stents at six time points within 180 days. (**a**) Base stent, (**b**) In-10% stent, (**c**) In-20% stent, and (**d**) In-40% stent.

The stress distributions of the stents (Base, In-10%, In-20%, and In-40%) at six time points (day 0, 30, 90, 120, 150, and 180) are shown in Figure 9. Generally, higher stress was observed at the supporting ring, while lower stress was observed at the connecting strut for all stents. The Base stent exhibited a higher stress level compared to the other three stents on day 0, and the maximum stress increased as the widening ratio decreased. This was because the lower radial stiffness, the more adequately the stent expanded under identical expansion pressure. The In-10% and In-20% stents shared similar stress distributions during degradation, and the In-20% stent exhibited a significant stress concentration (e.g., on day 120), which was attributed to its 28% higher radial stiffness compared to the In-10% variant. In contrast, the In-40% stent exhibited the lowest stress level throughout the entire degradation process, although it had the highest radial stiffness.

**Figure 10:**
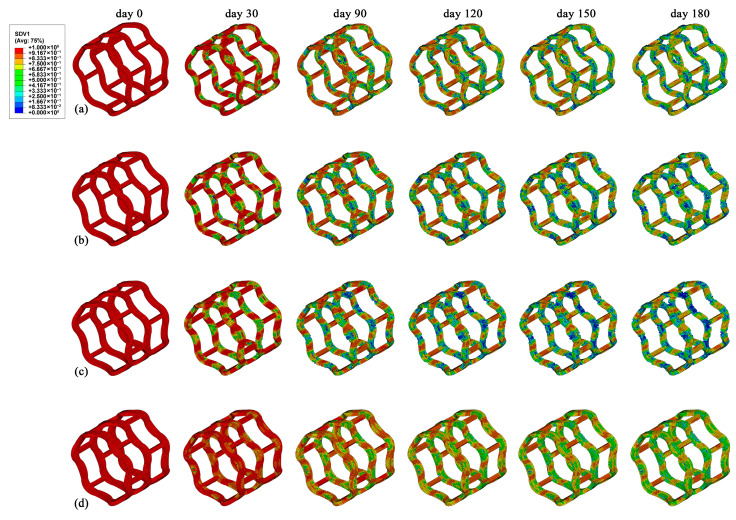
The β(t) distributions of the In-modified stents at six time points within 180 days. (**a**) Base stent, (**b**) In-10% stent, (**c**) In-20% stent, and (**d**) In-40% stent.

Figure 10 displays the evolution of β(t) distributions over six time points for the In-modified stent configurations. Generally, elements situated along the ring bends exhibited an accelerated degradation profile than those positioned on the interconnecting struts. This was due to the higher stress of the ring bends compared to other regions of the stent, suggesting that stress accelerated the degradation process by lowering the activation energy required for PLA hydrolysis [44,45]. The β(t) of the In-20% stent on the ring bends was lower than that of the other three stents, which is consistent with the higher stress level depicted in Figure 9c. By day 150, the middle ring of the In-20% stent was observed with extensive regions of β(t) lower than 0.1, which implies complete degradation of the elements. Specifically, the In-40% stent exhibited the highest β(t) among all stents, which corresponds to its lower stress levels during the degradation process.

**Figure 11:**
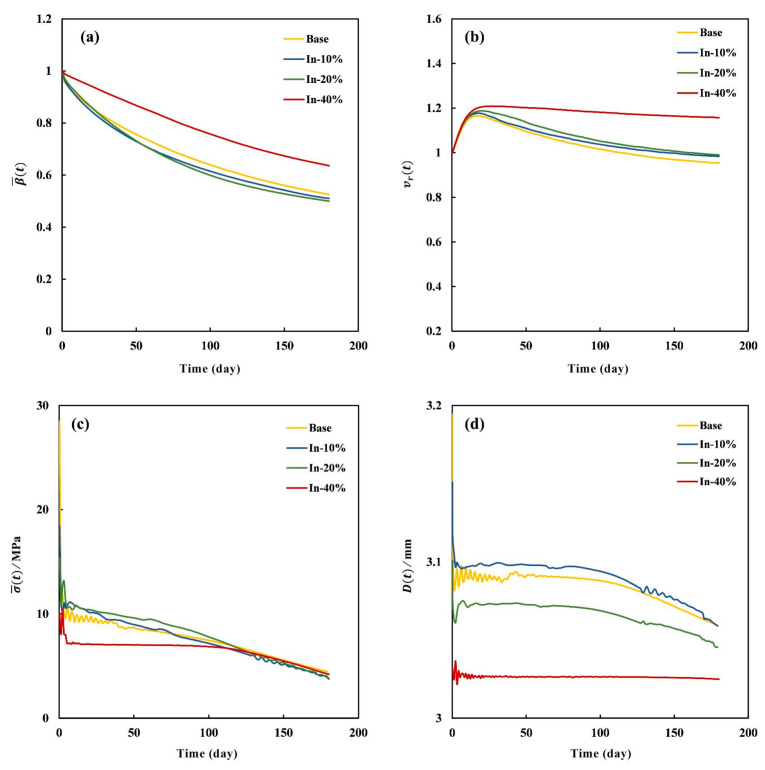
Evolution curves of four degradation indices for the In-modified stents (Base, In-10%, In-20%, and In-40% stents): (**a**) $\bar{\beta }\left(t\right)$, (**b**) $v_{r}\left(t\right)$, (**c**) $\bar{\sigma }\left(t\right)$, and (**d**) *D*(*t*).

The evolution curves of the four indices, $\bar{\beta }\left(t\right)$, $v_{r}\left(t\right)$, $\bar{\sigma }\left(t\right)$, and D(t), for the In-modified stents are shown in Figure 11. Generally, each of the four indices shared a similar decline tendency during degradation. For $\bar{\beta }\left(t\right)$, the In-10% and In-20% stents exhibited a decline similar to that of the Base stent, while the In-40% stent decreased at a significantly slower rate than the other three stents. On day 180, the $\bar{\beta }\left(t\right)$ values for the In-10%, In-20%, In-40%, and Base stents were 0.51, 0.50, 0.64, and 0.53, respectively. This was due to the lower stress of the In-40% stent, which decreased the degradation rate (Figure 9d). Incorporating the material’s swelling effect, *v*_r_ (*t*) reached a peak around day 20 before progressively decreasing, in agreement with experimental results reported in the literature [46]. Similarly to $\bar{\beta }\left(t\right)$, the $v_{r}\left(t\right)$ of the In-40% stent declined at a much slower rate. Although the $\bar{\beta }\left(t\right)$ of the In-10% and In-20% stents decreased more rapidly, their $v_{r}\left(t\right)$ values remained higher than the Base stent, which were 0.98, 0.99, and 0.95 on day 180, respectively. This indicates that the increase in material enhanced the stent’s volumetric integrity. The $\bar{\sigma }\left(t\right)$ values of the In-10% and In-20% stents were relatively higher than those of the other two stents before day 90, which is consistent with the stress distributions shown in Figure 9. Together with their higher decrease rates of $\bar{\beta }\left(t\right)$, this further suggests that stress accelerated the degradation process. The In-40% stent exhibited a markedly different trend in $\bar{\sigma }\left(t\right)$, remaining nearly constant until day 120 and subsequently declining to 4.2 MPa by day 180. The diameters in the peak expansion state were 3.19 mm, 3.15 mm, 3.11 mm, and 3.06 mm for the Base, In-10%, In-20%, and In-40% stents, respectively, and their counterparts in the post-expansion state were 3.11 mm, 3.11 mm, 3.07 mm, and 3.03 mm. The recoil% after expansion was calculated using the equation |Dexpan−Drecoil|/Dexpan×100% as 2.50%, 1.27%, 1.29%, and 0.98%, values that are comparable to the in vitro results of 2%-4.7% reported in literature, see Table 4 [47]. The discrepancies can be attributed to three aspects. Firstly, the Young’s modulus of the in vitro PLLA stent was 3.3 GPa, which is greater than that in the present study; secondly, the stents’ geometrical configurations are different, such as diameter, stent ring shape, etc.; finally, the displacement conditions are quite different. Despite these differences, the simulated results still showed good agreement with the in vitro experiment data. Moreover, the lowest expanding diameter of 3.06 mm for the In-40% stent accounted for the lowest stress level observed during the degradation process. On day 180, the diameters were 3.06 mm, 3.06 mm, 3.05 mm, and 3.02 mm for the Base, In-10%, In-20%, and In-40% stents, respectively, which indicates that the In-10% stent exhibited a better lumen diameter maintenance ability among the In- modified stents. The pathogenesis of in-stent restenosis involves several device-related mechanisms, including an inadequate radial strength, incomplete deployment, material fatigue failure, stent thickness, and incomplete lesion coverage [48,49]. Despite demonstrating a superior radial strength, the In-40% stent’s reduced maximum expansion diameter (3.06 mm vs. 3.19 mm for Base stent) indicates suboptimal deployment characteristics. This geometric constraint may elevate thrombosis risk due to incomplete stent apposition at vessel walls and increased flow disturbance [49]. Moreover, the prolonged degradation profile of the In-40% stent would significantly delay endothelialization, creating a critical window for in-stent restenosis [50].

The influence of widening ratio on stent degradation was further assessed through the evolution curves of the four degradation indices for the Out- and Both-modified stents (Supplementary Materials, Figures S1 and S2). The indices $\bar{\beta }\left(t\right)$, $v_{r}\left(t\right)$, and $\bar{\sigma }\left(t\right)$ exhibited similar trends in both stent groups, consistent with the behavior observed in the In-modified stents, except for the In-40% stent. These results suggest that as the widening ratio increased, the degradation accelerated while structural integrity improved, attributable to the greater material volume. Despite the performance of the In-40% stent, the Out-40% stent maintained the highest $v_{r}\left(t\right)$ value of 1.01, demonstrating an optimal structure integrity. The Out-modified stents maintained larger diameters than the Base stent throughout degradation, retaining a consistent diameter of 3.06 mm on day 180. The Both-modified stents maintained larger diameters than the Base stent until day 160, but decreased to lower values by day 180. These findings demonstrate that the Out-modified stents exhibited a superior lumen diameter maintenance capability compared to the other stent variants, despite their inferior radial stiffness.

**Figure 12:**
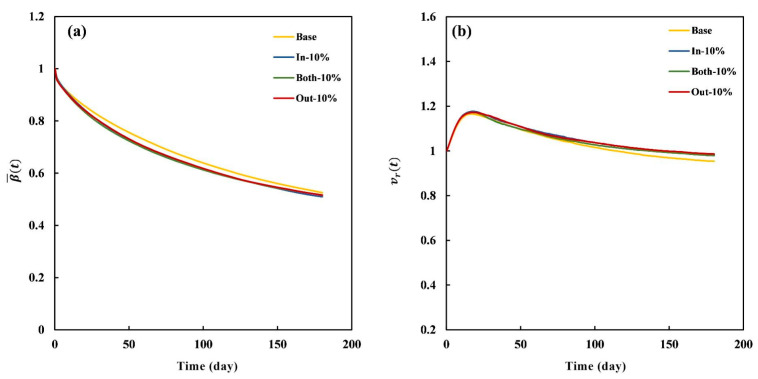

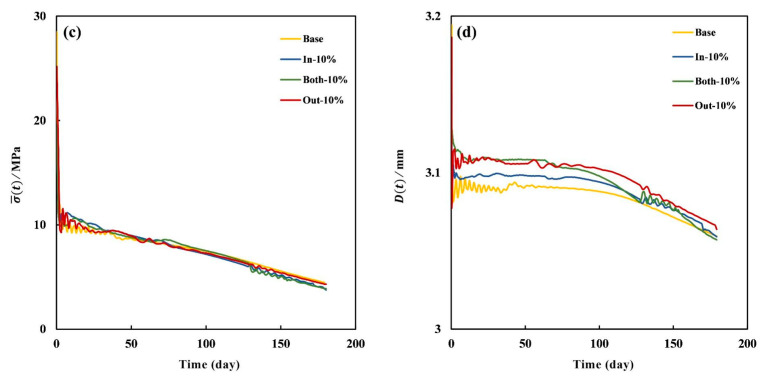
Evolution curves of four degradation indices for the 10%-modified stents (Base, In-10%, Both-10%, and Out-10% stents): (**a**) $\bar{\beta }\left(t\right)$, (**b**) $v_{r}\left(t\right)$, (**c**) $\bar{\sigma }\left(t\right)$, and (**d**) *D*(*t*).

To further investigate the influence of widening direction on the degradation properties, the evolution curves of the four degradation indices for the Base, In-10%, Both-10%, and Out-10% stents are displayed in Figure 12. Similar to the results in Figure 11, the $\bar{\beta }\left(t\right)$ values of the three modified stents were very close and relatively lower than that of the Base stent, which indicates a higher degradation rate for the modified stents. The $v_{r}\left(t\right)$ values of the three modified stents nearly overlapped and were slightly higher than that of the Base stent. This indicates that despite the accelerated degradation in the modified stents, the increased material concurrently reinforced their structural integrity. Moreover, the $\bar{\sigma }\left(t\right)$ values of the three modified stents were slightly higher than that of the Base stent, which indicates that stress accelerated the degradation of stent. The differences in stent diameter D(t) were more pronounced compared to the other three indices. In detail, the diameters in the peak expansion state were 3.19 mm, 3.15 mm, 3.17 mm, and 3.18 mm for the Base, In-10%, Both-10%, and Out-10% stents, respectively. During the degradation process, the Out-10% stent exhibited an optimal lumen maintenance ability with a relatively higher diameter, consistent with the results in Figure S1. Overall, these results demonstrate that widening direction had minimal influence on $\bar{\beta }\left(t\right)$, $v_{r}\left(t\right)$, and $\bar{\sigma }\left(t\right)$, while inducing significantly greater variation in D(t) across differently modified stents.

## 4. Conclusions

Optimizing the mechanical properties of stents enhances their long-term efficacy. Previous studies have shown that stent structure significantly influences a stent’s mechanical performance despite the degradation process [51,52,53]. In the present study, the mechanical and degradation properties of a modified commercial PLA stent were comprehensively evaluated to enhance its long-term efficacy. The results showed that both widening ratio and direction significantly affected the mechanical performance of the stent by increasing the radial stiffness, radial strength, and bending stiffness and minimizing the radial recoil%. Specifically, the In-modification strategy turned out to provide the best mechanical performance in terms of these parameters, despite the widening ratio. For the In-40% stent, its mechanical properties showed significant reinforcement compared to the Base stent: radial stiffness increased by 143.94%, radial strength increased by 104.17%, and bending stiffness increased by 63.43%, while radial recoil% decreased by 50.79%. Regarding the degradation properties, the widening ratio played a more significant role than the widening direction, as increased material enhanced the stent’s volumetric integrity and lumen diameter maintenance ability. However, an excessive increase in radial stiffness led to inadequate stent expansion, which may result in adverse clinical outcomes. Taking the In-40% stent as an example, its maximum expansion diameter was only 3.06 mm, resulting in the lowest overall stress level, prolonged degradation, and poorest lumen maintenance ability during degradation.

This study established a comprehensive framework for advancing biodegradable stent design considering three aspects. Firstly, the results elucidate the quantitative relationship between widening direction/ratio and stent performance, demonstrating that excessive widening (>40%) induces incomplete deployment and prolonged degradation, which may result in in-stent restenosis. Secondly, these findings allowed to evaluate long-term degradation–remodeling coupling in coronary arteries, particularly comparing curved vs. straight segments. Finally, the proposed designs provide a foundation for physical stent fabrication by computational optimization. Recent advances in additive manufacturing (AM), such as rotating mandrel-assisted AM, could realize these complex geometries even though challenges remain in maintaining a sub-50 µm precision while achieving clinical-grade mechanical properties [8]. Future work should couple the current computational framework with experimental manufacturing validation.

The present study has some limitations: (1) Only the bending regions of the support rings were parametrically designed, while other important geometric parameters, such as the number of connecting struts, strut configurations, pitch, and connector angle, were not considered. (2) More factors potentially influencing PLA degradation should be included, such as pH value and layered vessels. (3) The vascular geometry was modeled as a straight cylindrical tube with a uniform wall thickness, neglecting physiological features such as tapering, branching, and plaque formations. (4) This study did not experimentally validate the proposed designs. Although AM is a promising approach, its feasibility for our specific geometry modifications requires further investigation, particularly regarding dimensional accuracy and fatigue performance. Despite these limitations, the current findings still provide valuable insights for the structural design of PLA stents.

## Figures and Tables

**Table 1:** Material parameters.

Parameters	Parameter Symbols	Value	Unit
Young’s modulus of PLA	*E*	3000 [29]	MPa
Yield strength of PLA	$\sigma_{yield}$	50 [30]	MPa
Poisson’s ratio of PLA	ε	0.3 [31]	-
Elastic constants of vessel	$C_{10}$	1.02 [27]	MPa
	$C_{01}$	0.71 [27]	MPa
Elastic constants of balloon	$C_{10}$	1.07 [28]	MPa
	$C_{01}$	0.71 [28]	MPa

**Table 2:** Radial mechanical performances of all stents.

Stent	Radial Stiffness (N/mm)	Radial Strength (N/mm)
Base	34.32	0.24
In-10%	51.91	0.33
In-20%	57.57	0.36
In-40%	83.72	0.49
Both-10%	43.74	0.28
Both-20%	48.75	0.32
Both-40%	64.34	0.40
Out-10%	37.19	0.25
Out-20%	40.10	0.27
Out-40%	44.47	0.30

**Table 3:** Bending stiffness of all stents.

Stent	Bending Stiffness (N·mm^2^)
Base	3.61
In-10%	5.13
In-20%	5.27
In-40%	5.90
Both-10%	4.91
Both-20%	5.10
Both-40%	5.63
Out-10%	4.68
Out-20%	5.10
Out-40%	5.58

**Table 4:** Simulated recoil% of the In-modified stents after expansion vs. in vitro experimental results.

Stent	Material	D_expan_ (mm)	D_crimp_ (mm)	Recoil%
Base	PLA	3.19	3.11	2.50
In-10%		3.15	3.11	1.27
In-20%		3.11	3.07	1.29
In-40%		3.06	3.03	0.98
Wang et al. [47]	PLLA	3.70	-	~2.00
		3.92	-	~2.00
		3.10	-	4.70

## Data Availability

The data that support the findings of this study are available within the article.

## Supplementary Materials

The following supporting information can be downloaded at: https://www.mdpi.com/article/10.3390/jfb16070248/s1, Figure S1: Evolutions of the degradation indices of the four stents (Base, Out-10%, Out-20%, Out-40% stents): (**a**) $\bar{\beta }\left(t\right)$, (**b**) $v_{r}\left(t\right)$, (**c**) $\bar{\sigma }\left(t\right)$, and (**d**) *D*(*t*); Figure S2: Evolutions of the degradation indices of the four stents (Base, Both-10%, Both -20%, Both -40% stents): (**a**) $\bar{\beta }\left(t\right)$, (**b**) $v_{r}\left(t\right)$, (**c**) $\bar{\sigma }\left(t\right)$, and (**d**) *D*(*t*); Table S1: Geometric parameters of stents; Table S2: Element numbers of all stents in three simulations; Table S3: Stent diameters at different states during crimping simulation.
