# Supplementary material for: Influence of Stent Structure on Mechanical and Degradation Properties of Poly (Lactic Acid) Vascular Stent
Source: J Funct Biomater. 2025 Jul 6;16(7):248. doi: 10.3390/jfb16070248 (PMC12295491; doi:10.3390/jfb16070248)
Supplement: Supplementary file 1 [file jfb-16-00248-s001.zip › jfb-3678520-supplementary.pdf]

Supplementary Table S1. Geometric parameters of stents.

Stent	Stent length (mm)	Strut width（mm）
Base	10.5	0.2
In-10%	10.5	0.22
In-20%	10.5	0.24
In-40%	10.5	0.28
Both-10%	10.52	0.22
Both-20%	10.54	0.24
Both-40%	10.58	0.28
Out-10%	10.54	0.22
Out-20%	10.58	0.24
Out-40%	10.66	0.28

Supplementary Table S2. Element numbers of all stents in three simulations.

Stent	Crimping and bending	Degradation
Base	172758	10512
In-10%	193245	10944
In-20%	205115	11725
In-40%	214784	11257
Both-10%	194709	12868
Both-20%	203544	12868
Both-40%	216021	12868
Out-10%	177809	13079
Out-20%	197368	14284
Out-40%	214008	14976

Supplementary Table S3. Stent diameters at different states during crimping simulation.

Stent	D_{crimp} (mm)	D_{recoil} (mm)	Recoil (%)
Base	1.4	1.640	17.143
In-10%	1.4	1.587	13.345
In-20%	1.4	1.527	9.066
In-40%	1.4	1.518	8.435
Both-10%	1.4	1.619	15.672
Both-20%	1.4	1.578	12.713
Both-40%	1.4	1.552	10.854
Out-10%	1.4	1.628	16.326
Out-20%	1.4	1.583	13.099
Out-40%	1.4	1.577	12.629

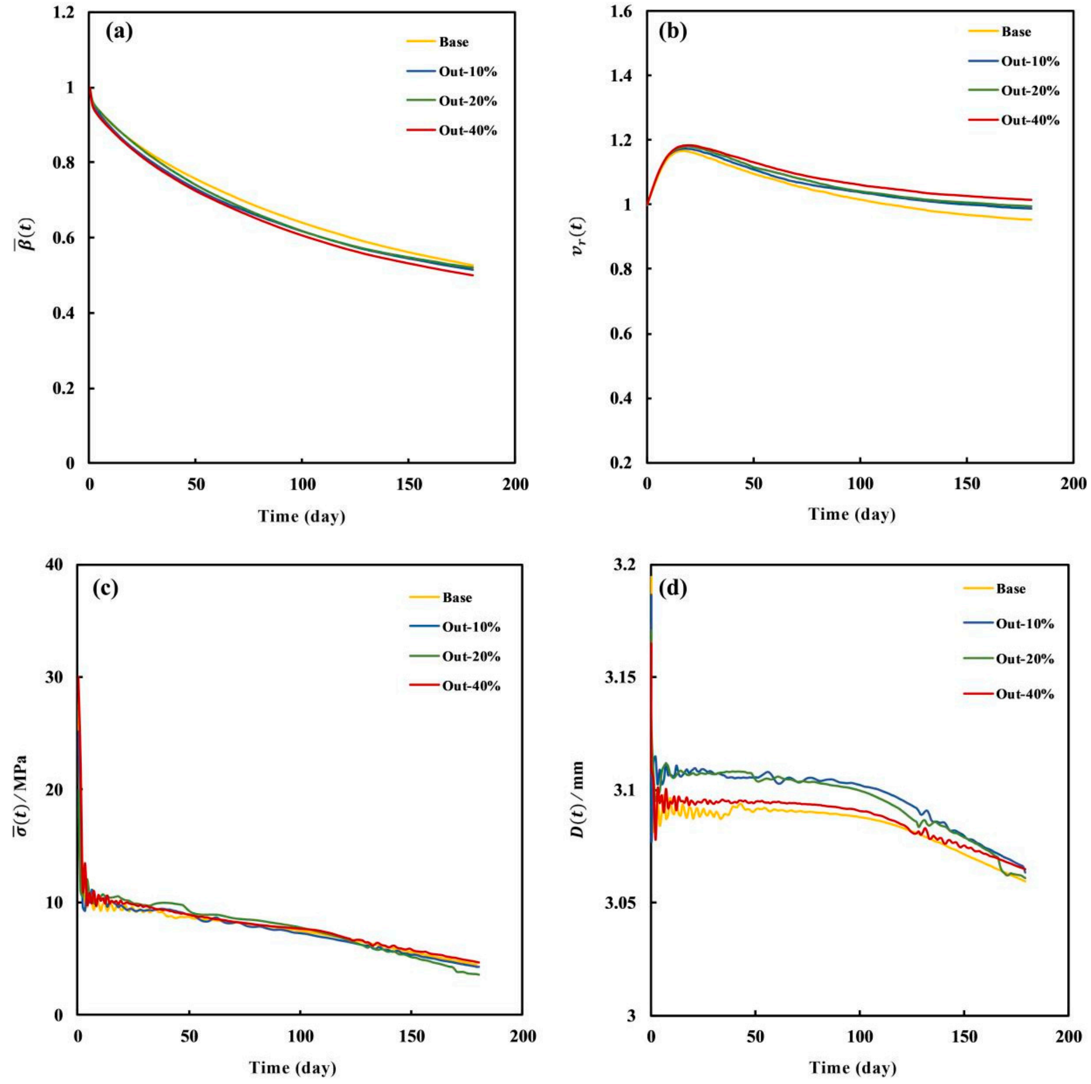

Supplementary Figure S1. Evolutions of the degradation indices of the four stents (Base, Out-10%, Out-20%, Out-40% stents): (a) $\bar{\beta}(t)$, (b) $v_{\mathrm{r}}(t)$, (c) $\bar{\sigma}(t)$, and (d) D(t).

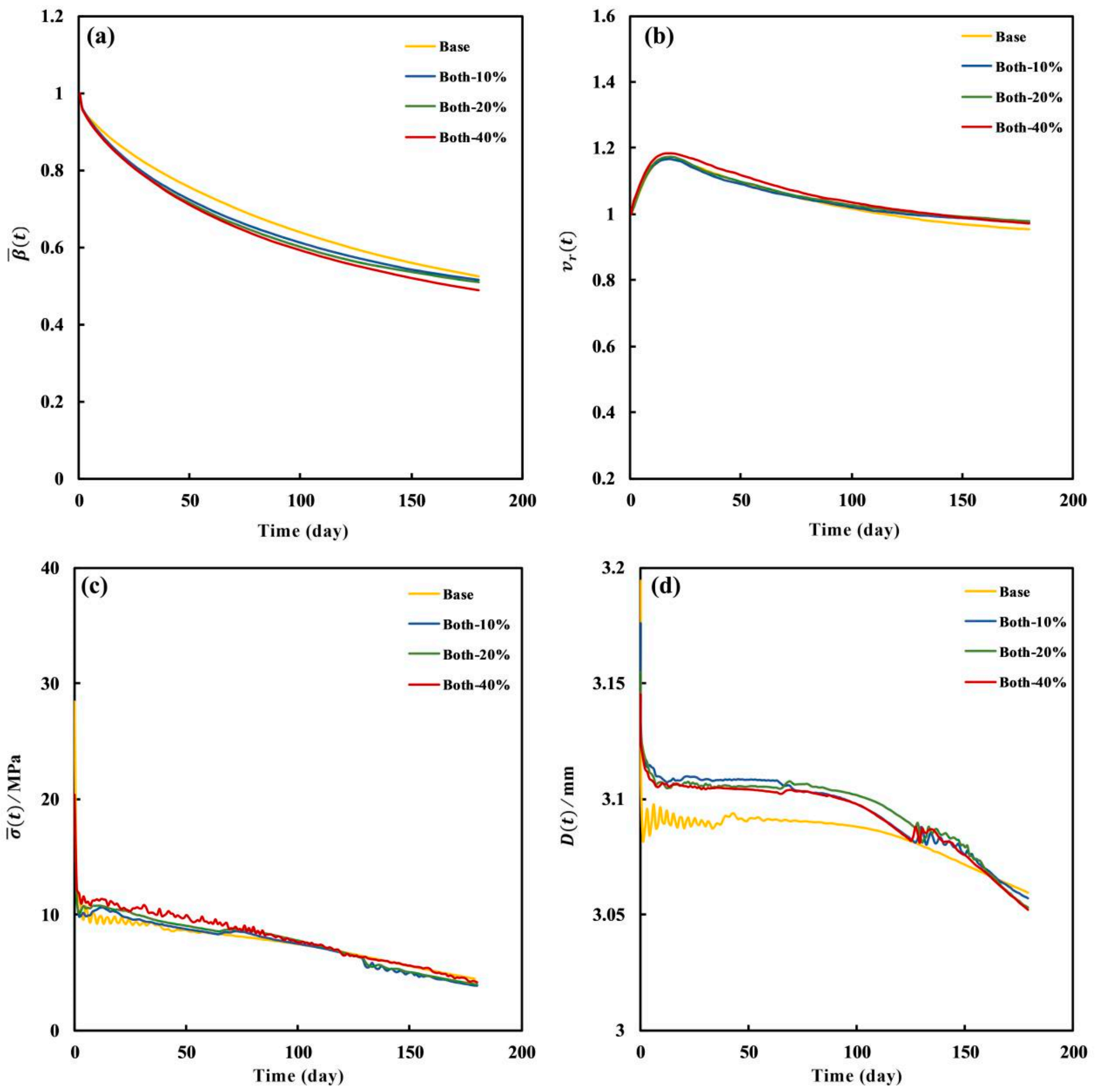

Supplementary Figure S2. Evolutions of the degradation indices of the four stents (Base, Both-10%, Both-20%, Both-40% stents): (a) $\beta(t)$, (b) $v_{\mathrm{r}}(t)$, (c) $\bar{\sigma}(t)$, and (d) D(t).
